# Peculiarities of Precocious Puberty in Boys and Girls With McCune-Albright Syndrome

**DOI:** 10.3389/fendo.2018.00337

**Published:** 2018-06-22

**Authors:** Domenico Corica, Tommaso Aversa, Giorgia Pepe, Filippo De Luca, Malgorzata Wasniewska

**Affiliations:** Unit of Pediatrics, Department of Human Pathology of Adulthood and Childhood, University of Messina, Messina, Italy

**Keywords:** McCune-Albright Syndrome, peripheral precocious puberty, endocrine manifestation, therapy, gonadotropin-independent precocious puberty, gonadal function

## Abstract

McCune-Albright Syndrome (MAS; OMIM # 174800) is a rare, sporadic disease caused by a post-zygotic, activating mutation in the guanine-nucleotide binding protein α-subunit (*GNAS1*) gene. MAS is characterized by the clinical triad of polyostotic fibrous dysplasia of bone, *café-au-lait* skin pigmentation and peripheral precocious puberty. However, clinical presentation is highly variable depending on mosaic tissue distribution of mutant-bearing cells. Precocious puberty is the most common endocrine manifestation of MAS and is often the presenting, and sometimes the only, clinical sign of MAS. Due to the very low prevalence of MAS, data on course of precocious puberty, effectiveness of treatments and gonadal function during post-pubertal period are lacking. Our knowledge on this issue derives essentially from case reports and small cohorts of patients. The aim of this review is to report all available literature data on clinical aspects, therapeutic management and outcomes of precocious puberty in children with MAS. A systematic research was carried out through MEDLINE via PubMed, EMBASE, Web of Science, Semantic Scholar, Cochrane Library.

## Introduction

McCune-Albright syndrome (MAS; OMIM # 174800) is a rare, sporadic disease caused by a post-zygotic, somatic, activating mutation in the guanine-nucleotide binding protein α-subunit (*GNAS1*) gene, encoding for the sub-unit α of the regulatory G-protein (G_s_α). Estimated prevalence ranges between 1/100,000 and 1/1,000,000, but reliable data on MAS prevalence are not available (1).

MAS is classically defined by the clinical triad of polyostotic fibrous dysplasia of bone, skin hyperpigmentation (*café-au-lait* spots) and precocious puberty (PP). Post-zygotic mutation determines a mosaic distribution of mutant-bearing cells resulting in a highly variable clinical presentation depending on the distribution of the affected cells and the specific tissues involved in the mosaicism.

Consequently, *GNAS1* mutation analysis has limited usefulness; genetic analysis on leukocytes exhibits positive results in only 8–46% of cases, and analysis of affected tissue, reaching a reliability of up to 90%, often resulted too invasive. Thereby, diagnosis is based primarily on clinical picture (2, 3). PP is the most common endocrine manifestation of MAS and is often the presenting, and sometimes the only, clinical sign of MAS.

The aim of this review is to report all available literature data on clinical aspects, therapeutic management and outcomes of PP in children with MAS. A systematic research was carried out through MEDLINE via PubMed (http://www.ncbi.nlm.nih.gov/pubmed/), EMBASE, Web of Science, Semantic Scholar, and Cochrane Library, based on the following keywords: “McCune Albright Syndrome” AND “precocious puberty.” We took original articles, case reports and systematic reviews of literature into consideration.

PP in MAS is usually a form of peripheral precocious puberty (PPP), independent from gonadotropin stimulation (4, 5). However, progression from PPP to central PP (CPP), in particular when bone age is advanced beyond 11 years, has been documented in girls (6–8).

The majority of MAS patients with PPP reported in literature are females, and there are only few reports on males (9). In a cohort of 113 subjects with MAS, PPP was diagnosed in 91% of girls and in no boys; however, boys were significantly less numerous: 15 vs. 98 (2). In an Italian cohort of 26 MAS children (10 boys and 16 girls), a significantly higher prevalence of PPP in girls than in boys (87.5 vs. 40%) has been reported (10). Rey et al. (11) suggested that such a dimorphism in the prevalence of precocious gonadal hyperactivity may be due to ovarian granulosa and theca cell populations' competence to produce steroids; activating mutation of *GNAS1* in any may determine a precocious, abnormal increase of steroid production. Conversely, in testes, only Leydig cells can produce androgens and, therefore, PPP in MAS males may manifest only when the somatic activating mutation of *GNAS1* involves Leydig cells but not in the cases where only Sertoli cells carry the mutation.

Considering that MAS-related PPP may have a different presentation and evolution in the two sexes, its clinical picture will be described separately in girls and boys.

## PPP in girls

PPP in girls with MAS is often determined by an autonomous ovarian secretion of estrogen from large, frequently unilateral, estrogen-producing ovarian cysts, which are the morphological manifestation of the autonomous hyperactivation of follicular cells, leading to local estrogen hypersecretion (12). The initial manifestation is usually vaginal bleeding, which may be either isolated or associated with development of breast tissue (Tanner stage II—III) and enhanced growth and bone age maturation rate (13); however, the natural history of PPP in girls with MAS is extremely variable. PPP onset can occur from neonatal period to early childhood, but evolution over time is highly unpredictable. Many girls have extended periods of quiescence after the first signs, characterized also by the involution of breast tissue; others have frequent episodes of vaginal bleeding and progression of pubertal development, with an increased risk of impaired final height (FH) prognosis (14).

Furthermore, due to the very low prevalence of this syndrome, data on the course of ovarian hyperfunction in adolescence and early adulthood with MAS are scarce. In their paper, Matarazzo et al. demonstrated the persistence of an intermittent autonomous ovarian hyperactivity at least 1 year after therapy withdrawal in 80% of female adolescents and young adults with MAS (13). Similar results have been reported by other authors in smaller cohorts of patients (15–17).

Sotomayor et al. (18) evaluated ovarian function in a cohort of 8 adolescents and young-adult MAS girls, with completed pubertal development and a history of untreated PPP, compared to healthy matched controls. These authors, comparing 28 and 57 consecutive menstrual cycles in MAS patients and controls respectively, demonstrated a significantly lower ovulatory rate among MAS girls. However, cases of MAS women that ultimately develop regular menses and have undergone normal pregnancies have been reported (19).

Typical biochemical findings of PPP include high estradiol levels, which are increased 2–3-fold more than expected in pre-pubertal age and are associated with suppressed gonadotropins. Pelvic ultrasound usually shows a unilateral ovarian cyst, which may have a mixed cystic and solid composition or a hemorrhagic aspect. These ultrasound findings overlap with those of juvenile granulosa cell tumors, and, when ovarian cyst is the only sign at the onset of MAS, patients may be at risk of an unnecessary oophorectomy.

### Management and therapeutic strategies

Management of PPP in females with MAS may require a pharmacologic intervention to prevent progression of pubertal development and an early epiphyseal fusion. Several different pharmacological strategies have been utilized (Table 1). However, although some drugs have been partially effective, the optimal pharmacologic treatment of PP in girls with MAS has not been identified.

**Table 1:** Available data on pharmacologic management of PPP in girls with MAS.

**Drugs**	**Effects of therapy**	**N. of patients (range of age at the beginning of the treatment)**	**References**
**Aromatase Inhibitors**	**First generation: testolactone**		
	- Unable to slow SMR and improve FH	12 (1.8–7.8 year)	(22)
	- Frequent daily administrations were needed	2 (6.2 and 6.5 year)	(23)
	**Second generation: fadrozole**		
	- Unable to block estrogen synthesis	16 (3.2–9.7)	(24)
	- Adverse inhibiting effect on both cortisol and aldosterone biosynthesis reported		
	**Third generation:**		
	**Anastrozole**	26 (3.2–11.0 year)	(25)
	- Unable to stop vaginal bleeding nor slow SMR	1 (3.9 year)	(26)
	**Letrozole**		
	- Able to decrease growth velocity and SMR	9 (3.3–8.1 year)	(27)
	- Able to stop vaginal bleeding	3 (3–5 year)	(28)
	- One episode of ovarian torsion reported	28 (1.5–8.3 year)	(29)
**Tamoxifen**	- Reduced vaginal bleeding	28 (2.9–10.9 year)	(30)
	- Decreased growth velocity and SMR.	1 (5.5 year)	(31)
	- Enlargement of ovarian and uterine volume reported	8 (0.3–3.7 year)	(32)
**Fulvestrant**	- Reduced vaginal bleeding and SMR	30 (1.7–8.5 year)	(33)
**Progestins (Medroxyprogesterone acetate)**	- Able to stop vaginal bleeding	5 (0.3–4.7)	(39)
		1 (1.5 year)	(36)
**Anti-androgens (Cyproterone acetate)**	- Unable to reduce SMR	1 (4.3 year)	(37)
		5 (3.9–6.3 year)	(38)
		1 (6 year)	(34)
**Ketoconazole**	- Reduced vaginal bleeding and SMR.	2 (7.4 and 7.11 year)	(23)
	- Unable to halt occurrence of ovarian cysts.		

*N, Number; FH, Final height; SMR, skeletal maturation rate; yr, year; BA, bone age*.

Aromatase inhibitors (AIs) directly interfere with estrogen biosynthesis, by binding the aromatase enzyme reversibly and preventing the conversion of androgens to estrogens, thus reducing the serum levels of estrogens. Three generations of AIs have been proposed for the treatment of PPP in MAS, however, they have not demonstrated a good level of efficacy. A first-generation AI, i.e., testolactone, although initially appearing effective in some case reports (20, 21), has been unable to induce any significant improvement in predicted FH by decelerating skeleton maturation rate in a long-term study (22) and adverse effects, such as nasal bleeding, recurrent vomiting and extreme behavior changes have been reported (23).

Fadrozole, a second-generation AI, which was employed in a cohort of 16 girls with MAS and PPP, was unable to block estrogen synthesis; furthermore, it also exhibited an adverse inhibiting effect on both cortisol and aldosterone biosynthesis (24).

Letrozole and anastrozole, third generation AIs, showed variable results. According to a study by Mieszczak et al. (25), anastrozole does not seem to be able to stop vaginal bleeding nor attenuate rates of skeletal maturation and linear growth.

Alves et al. (26) reported that anastrozole, administered for 36 months to a 3.9 year-old girl with PPP, induced the following phenomena: suppression of breast tissue growth, normalization of growth velocity and serum estradiol level, and regression of ovarian cysts. However, the patient exhibited an increase in uterine volume, an advancement of bone age and two episodes of vaginal bleeding during follow-up.

Feuillan et al. (27), in a cohort of 9 girls aged between 3 and 8 years, observed a significant decrease in growth velocity and skeletal maturation rate during letrozole treatment; however, these authors observed an episode of ovarian torsion and an increase of mean ovarian volume and estrogen levels during follow-up, raising some concerns regarding the safety of this drug.

In a small case series, letrozole was effective in preventing pubertal progression, even though in one patient this therapy was discontinued due to the appearance of fatigue (28). In a retrospective analysis involving a cohort of 28 MAS girls with PPP, Estrada et al. (29) reported letrozole long-term safety and efficacy, in terms of final height improvement, vaginal bleeding episode disappearance/reduction, skeletal maturation rate and growth velocity decrease; changes in uterine size or ovarian volumes were not documented.

An alternative to AIs is represented by tamoxifen, a selective estrogen-receptor modulator, which showed encouraging results in MAS patients with PPP. Eugster et al. (30), in a cohort of 25 subjects, demonstrated a reduction of vaginal bleeding and a significant decrease of growth velocity and skeletal maturation rate after 12 months of tamoxifen treatment. Nevertheless, these authors reported an enlargement of ovarian and uterine volume, probably due to the estrogen agonistic effects of tamoxifen, that raised some concerns on the long-term safety of this therapy (30, 31). Recently, de G Buff Passone et al. (32) carried out a retrospective long-term study that evaluated the effects of tamoxifen in girls with PPP and MAS for a mean period of 8.4 years. These authors did not observe any adverse effects, although the improvement of predicted FH was less significant than that expected.

A pure estrogen receptor antagonist, fulvestrant, has been proposed by Sims et al. (33) in girls with PPP and MAS. In a prospective study, these authors demonstrated good tolerability and promising efficacy in decreasing vaginal bleeding and rates of skeletal maturation after a 12-month treatment. Progestins, such as medroxyprogesterone acetate, and anti-androgens, such as cyproterone acetate (34) and ketoconazole (23), have also been used in the past (35, 36); nevertheless, even though they were effective in arresting vaginal bleeding, these therapies are not able to affect either skeletal maturation or FH (37–39).

Pharmacologic treatments are the first-choice therapy for PPP in MAS, however, when drugs are not effective a surgical approach has been considered.

Gesmundo et al. (40) performed a trans-umbilical laparoscopic assisted ovarian cystectomy in four MAS girls with PPP, in which pharmacologic treatment had not been effective. In three patients, a prolonged remission from biochemical and clinical signs of hyperestrogenism was reported at 5 years, 1 year and 18 months of follow-up, respectively. In one out of four patients, vaginal bleeding and a cyst, in the same ovary in which cystectomy had been performed, recurred.

In a case report, ovarian cystectomy performed in a 3-year-old MAS girl resulted in PPP signs arrest over almost 4-years of follow-up (41). In another study, resolution of PPP symptoms in MAS girls after ovariectomy has been described; however, a 5-month follow-up was reported (37).

In contrast, some case reports demonstrated recurrence of PPP symptoms in MAS girls in spite of cystectomy or oophorectomy (42, 43).

In a cohort of nine girls, a recurrence of PPP symptoms in three out of four girls who underwent salpingo-oophorectomy was reported (43).

Other studies reported outcomes of a surgical approach in adult MAS women with ovarian cysts. Laven et al. (44) reported a case of a 22-year-old MAS woman with irregular menses and bilateral ovarian cysts. *GNAS1* mutation was demonstrated only in the right ovarian tissue. Regular menses and reproductive function have been restored through right ovariectomy.

Similarly, unilateral ovariectomy determined regular menses recovery and abdominal pain resolution in a 33-year-old MAS woman with unilateral ovarian cysts (45).

In another 22-year-old MAS girl, unilateral ovariectomy, performed to restore regular menses, revealed a borderline epithelial ovarian tumor, therefore these authors suggested that early, prolonged acyclic hyperestrogenemia may enhance the risk of this estrogen-dependent cancer (17).

Furthermore, the observation of a virilizing sclerosing-stromal tumor of the ovary in a woman with MAS extends the clinical spectrum of ovarian pathology in MAS (46). However, the mechanisms causing this ovarian tumor remain unclear, even if the activating G_s_α mutation has been implicated in the pathogenesis of some gonadal tumors.

Although some case reports suggested effectiveness of the surgical approach in MAS patients with ovarian cysts, due to limited and contrasting evidence available, especially in children, surgical interventions should be considered only in those cases with significant abdominal pain or at risk for ovarian torsion and in selected adult patients.

To sum up, PPP in girls with MAS is frequently determined by estrogen-producing ovarian cysts and isolated vaginal bleeding may be the first manifestation; however, its clinical course is highly variable. Surgical resection of cysts or oophorectomy are not always effective in PPP because of the possible recurrence of cysts in the remaining ovarian tissue and it should be considered only in selected patients with PPP, after varying pharmacologic treatment failure. Although different pharmacologic interventions seem promising, more prolonged studies are needed to establish safety and effectiveness.

## PPP in boys

PPP is a rare condition in boys with MAS, which is characterized by enlargement of testes and signs of sexual precocity, usually associated with serum testosterone levels in the pubertal range and suppressed gonadotropin levels (10).

Macroorchidism, due to autonomous hyperfunction and hyperplasia of Sertoli and Leydig cells, may be monolateral (47, 48) and represents an early, isolated clinical manifestation of MAS (49, 50). Testicular autonomous hyperfunction in MAS may also be restricted to Sertoli cells, due to a localized *GNAS1* mutation. In these cases, with isolated hyperplasia and hyperfunction of Sertoli cells, testis enlargement has been found to be associated with increased levels of inhibin B and anti-Mullerian hormone and pre-pubertal values of serum testosterone and gonadotropins. In these cases, macroorchidism is not accompanied by signs of sexual precocity (11, 49, 51, 52).

An additional manifestation of MAS in boys is testicular microlithiasis (TM), i.e., an ultrasonography picture whose prevalence in the pediatric general population has been estimated to range around 5% (53), whereas in boys with MAS it has been reported to be significantly higher, that is, between 30 and 62% (54, 55). The clinical implication of such an increased prevalence of TM in MAS boys, however, has not been clarified to now (55). Apart from TM, other testicular lesions may possibly be observed in MAS boys. Boyce et al. (55), in a large cohort of children and adult males, documented TM in 30%, focal hyper–and hypoechoic lesions in 49 and 30%, respectively, diffuse heterogeneity and focal calcifications in 47 and 11%. Poorly differentiated embryonal cell testicular tumor has been demonstrated only in a 28-year-old patient, who, 5 years after diagnosis, developed a seminoma in the contralateral testicle. Longitudinal follow-up (mean 4.6 years) revealed no or minimal progression in size of testicular lesions in 73% of cases, and moderate or significant progression in 22%; none developed local invasion or metastases (55).

Due to the rarity of MAS, data on natural history of sexual development and adult fertility in males with MAS and PPP are scarce. De Luca et al. (48) described the long-term follow-up of a boy with MAS, from diagnosis of PPP at the age of 2.9 years to the age of 18 years, when sexual development was completed, and FH was reached. Those authors documented persistently elevated sexual steroid levels with suppressed gonadotropin levels during the entire follow-up, a finding which was compatible with the persistence of the autonomous testicular hyperfunction over time. Furthermore, they documented an effective spermatogenesis, with the evidence of germ cells maturation in the histologic testis samples, despite sustained and marked FSH suppression.

### Management and therapeutic strategies

Aims of treatment in boys with MAS-related PPP are to decelerate androgenization evolution and growth velocity, to prevent epiphyseal fusion and ultimately improve FH. There is a limited availability of published data on treatment of PP in boys with MAS (Table 2).

**Table 2:** Available data on pharmacologic management of PPP in boys with MAS.

**Drugs**	**Effects of therapy**	**N. of patients (range of age at the beginning of the treatment)**	**References**
**Testolactone** + **Flutamide**	- Reduced growth velocity and testis volume.	1 (8.5 year)	(56)
	- Testolactone needed frequent daily administrations		
	- Flutamide associated with hypertransaminasemia and neutropenia	1 (5 year)	(55)
**Testolactone** + **Spironolactone**	- Improved BA/chronological age ratio and predicted FH	3 (3–8 year)	(55)
**Letrozole** + **Spironolactone**		1 (8 year)	
**Letrozole** + **Spironolactone** + **Flutamide**	- PPP evolution and growth outcomes not reported	1 (6 year)	(50)
**Anastrozole** + **Bicalutamide**	- Normalized growth velocity	1 (4.6 year)	(57)
	- Reduced androgenization		
	- Stabilized testicular volume		
**Ketoconazole** + **Cyproterone Acetate**	- Decelerated growth velocity	1 (4.6 year)	(58)
	- Reduced both testicle size and testosterone and free testosterone serum levels		
	- Improved FH		

*N, Number; BA, Bone Age; FH, Final height; yr, year; PPP, peripheral precocious puberty*.

Zacharin et al. (56) reported reduction of growth velocity and testis volume in a boy with MAS and PPP, when treatment with testolactone and flutamide was undertaken; however, flutamide had to be withdrawn after 3 months, due to the concomitant finding of both hypertransaminasemia and neutropenia. Longitudinal data of 5 boys with MAS and PPP, treated with different combined therapy approaches (flutamide + testolactone in one patient; testolactone + spironolactone; spironolactone + letrozole, associated with leuprolide acetate when CPP occurred) demonstrated improvement in both bone age/chronological age ratio and predicted FH (55). However, the employment of testolactone is limited by the need of frequent daily administrations. Rustagi et al. (50) reported the case of a boy with MAS and PPP diagnosed at the age of 6, who was treated with letrozole, spironolactone and flutamide; however, PPP evolution and growth outcomes have not been described. In a case report, Tessaris et al. (57) showed efficacy and safety of combined therapy with third generation AIs and competitive androgen receptor blockers (anastrozole and bicalutamide, respectively), during a period of 49 months, in a MAS boy with PPP.

Recently, Messina et al. (58) described the case of a MAS boy with monolateral macroorchidism and PPP treated with an association of ketoconazole and cyproterone acetate, aiming to suppress testicular steroidogenesis and to inhibit the peripheral effects of testosterone and dihydrotestosterone. During the first 12 months of treatment, these authors reported a significant deceleration of growth velocity and reduction of both testicle size and testosterone and free testosterone serum levels. Two years later, because of CPP onset, leuprolide acetate depot was associated. This combined therapy enabled this patient to achieve a FH very close to that predicted at onset of treatment, without any side effects.

Few data are available on a surgical approach in MAS boys with PPP. Boyce et al. (55), in those subjects (age range from 14–54 years) who underwent total orchiectomy or testis-sparing partial orchiectomy, demonstrated 11 benign lesions (predominantly Leydig cell hyperplasia) and one malignant lesion. Therefore, these authors recommend a conservative approach to management of MAS-associated testicular lesions, and suggest testicular preservation, clinical and ultrasonographic close observation, as activating G_s_α mutation in MAS may be a predisposing factor for testicular malignancy.

To sum up, PPP in boys with MAS is generally characterized by bilateral macroorchidism associated with signs of sexual precocity; however, monolateral testicular enlargement may also be reported. Testicular lesions should be closely monitored by clinical and ultrasonographic evaluations. Surgical approach is not the first-choice treatment and should be considered for lesions that are rapidly progressive and/or locally invasive at clinical and ultrasonographic evaluations. Long-term studies are needed to verify efficacy and safety of the different therapeutic approaches.

## Conclusions

(1) MAS should be considered in the differential diagnosis of PPP in both sexes. (2) A gold standard treatment for MAS-related PPP has not yet been established. (3) Gonadal function during post-pubertal period does not seem to be impaired among the majority of MAS patients. (4) An autonomous gonadal hyperfunction may persist over time in both sexes.

## Author contributions

MW, FD, and DC conceived the review. DC, TA, and GP were involved in literature search. DC, MW, and FD were involved in writing of the manuscript. All authors approved the submitted version of the manuscript.

### Conflict of interest statement

The authors declare that the research was conducted in the absence of any commercial or financial relationships that could be construed as a potential conflict of interest.
